# L1CAM Reliably Distinguishes Low-Grade Oncocytic Tumor from Other Eosinophilic Renal Neoplasms: A Multicenter Immunohistochemical Study with Diagnostic Implications

**DOI:** 10.3390/cancers17152440

**Published:** 2025-07-23

**Authors:** Luciana Scuccimarri, Antonio d’Amati, Francesco Pierconti, Angela Santoro, Luigia Ciampi, Tiziana Montrone, Francesco Alfredo Zito, Giuseppe Lucarelli, Guido Rindi, Gian Franco Zannoni, Mauro Giuseppe Mastropasqua

**Affiliations:** 1 Anatomic Pathology Unit, Department of Precision and Regenerative Medicine—Ionian Area, University of Bari “Aldo Moro”, 70124 Bari, BA, Italy; luciana.scuccimarri16@gmail.com; 2 Anatomic Pathology Unit, General Regional Hospital F. Miulli, 70021 Acquaviva delle Fonti, BA, Italy; l.ciampi@miulli.itt; 3 Department of Translational Biomedicine and Neuroscience, University of Bari Medical School, 70124 Bari, BA, Italy; 4 Anatomic Pathology Unit, Department of Woman and Child’s Health and Public Health Sciences, Fondazione Policlinico Universitario Agostino Gemelli IRCCS, Università Cattolica del Sacro Cuore, 00168 Rome, RM, Italy; francesco.pierconti@unicatt.it (F.P.); angela.santoro@policlinicogemelli.it (A.S.); guido.rindi@unicatt.it (G.R.); gianfranco.zannoni@unicatt.it (G.F.Z.); 5 Anatomic Pathology Unit, Santissima Annunziata Hospital, 74121 Taranto, TA, Italy; tiziana.montrone@aslta.it; 6 Anatomic Pathology Unit, I.R.C.C.S. Istituto Tumori “Giovanni Paolo II”, 70124 Bari, BA, Italy; fazito@libero.it; 7 Urology, Andrology and Kidney Transplantation Unit, Department of Precision and Regenerative Medicine and Ionian Area-Urology, University of Bari “Aldo Moro”, 70121 Bari, BA, Italy; giuseppe.lucarelli@uniba.it; 8 Urology Unit, I.R.C.C.S. Istituto Tumori “Giovanni Paolo II”, 70124 Bari, BA, Italy

**Keywords:** L1CAM, low-grade oncocytic tumor, eosinophilic renal neoplasms, immunohistochemistry, renal tumor classification

## Abstract

**Introduction:** Low-grade oncocytic tumor (LOT) is a recently described renal neoplasm characterized by indolent clinical behavior, a small nested architecture, and distinctive immunophenotypic features. Its distinction from other eosinophilic renal tumors, such as oncocytoma, eosinophilic chromophobe renal cell carcinoma (E-chRCC), and eosinophilic vacuolated tumor (EVT), can be challenging due to overlapping features. The L1 cell adhesion molecule (L1CAM) is being increasingly recognized as a potential diagnostic marker for LOT. **Aims:** To evaluate the diagnostic performance of L1CAM in distinguishing LOT from morphologically and immunophenotypically similar eosinophilic renal neoplasms. **Methods:** A total of 54 eosinophilic renal tumors (10 LOTs, 22 oncocytomas, 18 E-chRCCs, and 4 EVTs) were retrospectively collected from five academic institutions and reclassified according to the 2022 WHO criteria. All cases underwent histopathologic review and immunohistochemical analysis for CK7, CD117, GATA3, cathepsin K, and L1CAM. **Results:** L1CAM showed strong membranous expression in all LOTs (100%) and was negative in oncocytoma, E-chRCC, and EVT, yielding 100% sensitivity and specificity. Traditional markers exhibited overlapping patterns among tumor types. **Conclusions:** Our findings confirm L1CAM as a highly sensitive and specific marker for LOT, effectively distinguishing it from other eosinophilic renal neoplasms. Incorporating L1CAM into diagnostic panels may enhance accuracy, particularly in challenging cases.

## Simple Summary

Kidney tumors with cells that appear pink under the microscope—called eosinophilic tumors—can be difficult to classify correctly. Some of these tumors are harmless, while others may require close monitoring or surgery. A rare type called low-grade oncocytic tumor looks similar to other kidney tumors, which makes diagnosis challenging, especially in small biopsies. In this study, we examined a group of kidney tumors that look alike but have different behaviors. We focused on a protein called L1CAM, which is found on the surface of some tumor cells. Our results showed that L1CAM was present only in low-grade oncocytic tumors and not in the other tumor types studied. This means that L1CAM could help pathologists more accurately identify this rare tumor type. Better diagnosis can help avoid unnecessary treatments or surgery for patients. This work also encourages future research to understand how this protein behaves in other kidney tumors. Using tools like this in everyday diagnostic practice may lead to more personalized and appropriate care for people with kidney tumors.

## 1. Introduction

The 2022 WHO Classification of Renal Tumors has refined the taxonomy of renal cell carcinoma (RCC) by integrating molecular data to delineate histological subtypes and define novel entities [1]. Eosinophilic renal tumors represent a heterogeneous group of neoplasms characterized by abundant eosinophilic cytoplasm, typically due to mitochondrial accumulation. This spectrum includes benign oncocytoma, chromophobe RCC (chRCC), and eosinophilic variants of other RCC subtypes [2]. The recent recognition of low-grade oncocytic tumor (LOT) and eosinophilic vacuolated tumor (EVT) has further expanded the differential diagnosis of these neoplasms [3]. Accurate classification of eosinophilic tumors is critical, particularly in limited biopsy samples, but can be challenging due to overlapping architectural and cytologic features. Immunohistochemical markers such as CK7, CD117, GATA3, and cathepsin K are commonly used, but often lack sufficient specificity [4]. Low-grade oncocytic tumor (LOT) has been proposed as a distinct renal entity with unique architectural (small nested pattern), cytological (perinuclear halos and irregular nuclear contours), and immunohistochemical features (CK7 positivity, CD117 negativity, and GATA3 expression). Its correct identification is essential due to its indolent behavior and benign clinical course. Immunohistochemistry remains the most important tool in the differential diagnosis of eosinophilic renal tumors despite its limited specificity. In this context, novel markers such as L1CAM may offer additional diagnostic value and help improve classification accuracy. L1 cell adhesion molecule (L1CAM) is a transmembrane glycoprotein, physiologically expressed in the basolateral membrane of principal cells in the renal collecting system and distal tubules Among renal tumors, its expression has been consistently reported in LOT, while it is typically absent in oncocytomas, E-chRCC, and EVT [5]. Rare cases of hybrid oncocytic/chromophobe tumors or SDH-deficient RCCs may also show L1CAM immunoreactivity, albeit with lower consistency [6,7]. This emerging pattern suggests both diagnostic and lineage relevance.

Although markers such as GATA3 and FOXI1 have been proposed to support a principal versus intercalated cell origin, L1CAM appears to demonstrate superior diagnostic accuracy in distinguishing LOT from E-chRCC [5,8]. Given the morphological and immunophenotypic overlap among eosinophilic renal tumors, we aimed to evaluate the diagnostic performance of L1CAM in differentiating LOT from oncocytoma, E-chRCC, and EVT in a multi-institutional cohort. We also assessed its utility in routine practice as a reliable ancillary marker in the classification of renal oncocytic tumors.

## 2. Materials and Methods

### 2.1. Case Cohort and Histopathologic Review

A retrospective, multicenter review was performed across five Italian academic institutions (University Hospital Policlinico of Bari, Italy; IRCCS Oncologic Institute “Giovanni Paolo II” of Bari, Italy; Regional General Hospital “Miulli” of Acquaviva delle Fonti, Bari, Italy; “Santissima Annunziata” Hospital of Taranto, Italy; University Hospital Policlinico IRCCS “Agostino Gemelli” of Rome, Italy) to identify eosinophilic renal tumors resected between 2016 and 2024. All samples were derived from surgical nephrectomy specimens and processed as formalin-fixed, paraffin-embedded (FFPE) tissue blocks. Included cases had a prior diagnosis of oncocytoma, E-chRCC, EVT, LOT, or oncocytic renal neoplasm of low-malignant-potential NOS. All available histologic slides and FFPE blocks were reviewed centrally by two uropathologists (F.P. and M.G.M.), with additional immunohistochemistry performed when necessary. Cases were reclassified according to the 2022 WHO criteria into four final diagnostic categories: oncocytoma, LOT, EVT, and E-chRCC. The study was approved by the Ethics Committee of the University of Bari (protocol no. 143/CE/2015) and conducted in accordance with the Declaration of Helsinki (1975, revised 2013).

### 2.2. Clinical and Morphologic Parameters

Clinical (age, sex, and type of surgery) and pathological (gross description, type of surgery, site and tumor size, and focality) data were obtained from the histological report.

Pathological tumor stage (pT) was reassigned according to the 8th edition of the AJCC Cancer Staging Manual, also reported in the 2022 World Health Organization Classification [1]. Although TNM staging is typically reserved for malignant tumors, we applied pT classification also to benign tumors (LOT, oncocytoma, and EVT) in order to enable standardized comparison across the cohort and with the existing literature.

Histomorphologic features were reviewed, and the following parameters were recorded: dominant tumor architecture, presence/absence of sharply demarcated areas of stromal edema, peri-nuclear clearing/halos, irregular nuclear contour, and any other unusual feature. The three dominant architectural patterns used for analysis included the following: “small nested” (small solid nests; oncocytoma-like), “classic chRCC” (solid, sheets-like, or broad trabecular with incomplete vascular septa), and “tubular” (tubular, alveolar, or tubulocystic growth).

### 2.3. Immunohistochemical Staining and Scoring Criteria

IHC was performed on 5 μm thick sections obtained from formalin-fixed, paraffin-embedded (FFPE) tissue utilizing antibodies for CK7 (clone *OV-TL 12/30, Dako; ready to use*), CD117 (*Polyclonal Rabbit Anti-Human, Dako; 1:100*), GATA3 (clone *L50-823, Diagnostic BioSystems, 1:75*), and Cathepsin K (clone *3F9, Vitro Master Diagnostica, 1:200*) as well as = L1 cell adhesion molecule (L1CAM) (clone *14.10, Biolegend, 1:200*). All immunohistochemical stains, including L1CAM, were performed centrally at the University Hospital Policlinico of Bari using standardized and validated protocol. Detection was carried out using the Dako EnVision™ FLEX detection system (Agilent Technologies, Santa Clara, CA, USA), following the manufacturer’s instructions.

Staining for each antibody was scored as diffuse, focal/patchy, or negative (defined as <5% scattered positive cells). L1CAM staining was considered positive only when showing distinct and diffuse membranous labeling.

Immunostained slides were reviewed independently by two pathologists (A.d.A., M.G.M.). In the event of discordant interpretation, a consensus diagnosis was reached.

Sensitivity, specificity, and 95% confidence intervals were calculated using the Clopper–Pearson exact method for binomial proportions.

Representative images were acquired using a light microscope equipped with a digital camera, and all magnifications, specified in the figure legends, (10×, 25×, 50×, 100×, and 200×) refer to objective lens power.

Table 1 summarizes the criteria used for the immunohistochemical evaluation.

## 3. Results

A total of 54 eosinophilic renal tumors were included following centralized histopathologic review and reclassification according to the 2022 WHO criteria [1]: 10 LOTs, 22 oncocytomas, 18 eosinophilic chromophobe RCCs (E-chRCCs), and 4 eosinophilic vacuolated tumors (EVTs).

The clinicopathologic features of these four cohorts are summarized in Table 2.

### 3.1. Low-Grade Oncocytic Tumor Cohort

The LOT cohort consisted of 10 cases from 10 patients. The median age of patients was 70 years (58–77) at diagnosis, with a clear predominance in females (M/F = 1:9). One case was reclassified from E-chRCC to LOT after central review and additional IHC. All cases were confined within the kidney (pT1-T2).

Grossly, the tumors were well-demarcated and had tan-yellow or brown cut surfaces with hemorrhagic areas. Focal cystic changes were seen in a subset of cases.

**Figure 1:**
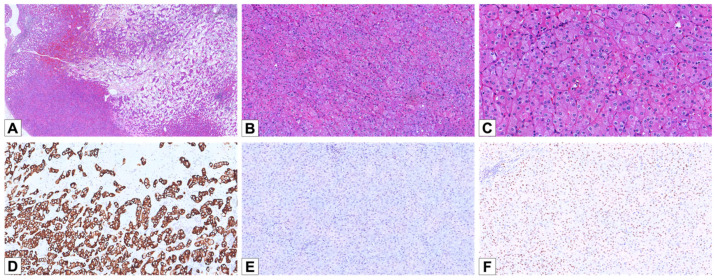
Histopathological and immunohistochemical features of LOT. (**A**) Hematoxylin and eosin staining of a LOT case, demonstrating the characteristic “small nested” architectural pattern with transition to stromal edema or hemorrhage (HE, 30×). (**B**) The tumor cells exhibit a small nested architecture with eosinophilic cytoplasm and prominent perinuclear halos (HE, 100×). (**C**) Nuclei show irregular nuclear contours and perinuclear halos (HE, 200×). (**D**) Diffuse and strong positive staining for CK7 (100×). (**E**) Negativity for CD117 (100×). (**F**) Weak/moderate nuclear positivity for GATA3 (100×).

Microscopically, all cases displayed compact, small nests arranged in a predominantly solid growth pattern; seven cases (70%) showed abrupt transition to areas of stromal edema or hemorrhage, with tumor cells arranged in cords, trabeculae, or tubules (Figure 1A). Neoplastic cells showed granular to densely eosinophilic cytoplasm, with prominent perinuclear halos and round-to-oval nuclei often exhibiting smooth to mildly irregular contours (Figure 1B,C).

By IHC, eight LOT cases (90%) showed diffuse positivity for CK7 (Figure 1D), and two cases showed focal/patchy positive staining. CD117 expression was absent in nine cases and focal in one case (Figure 1E). All LOTs were positive for GATA3, with diffuse staining in five cases and focal staining in the remaining five. Additionally, all cases were negative for cathepsin K.

### 3.2. Oncocytoma Cohort

**Figure 2:**
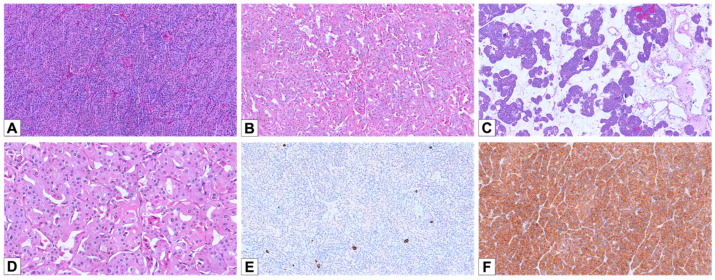
Histopathological and immunohistochemical features of oncocytoma. (**A**) A case of oncocytoma displaying a solid/trabecular architecture (HE, 100×). (**B**) A different case, showing a tubular/alveolar pattern (HE, 100×). (**C**) An oncocytoma with a prevalent solid/trabecular architecture and focal areas composed of small nests in an edematous background (HE, 50×). (**D**) Oncocytoma cells slightly displaying irregular nuclear contours and thin perinuclear halos (HE, 200×). (**E**) Negativity for CK7, except for rare scattered and isolated cells (100×). (**F**) Diffuse cytoplasmic positivity for CD117 (100×).

The oncocytoma cohort consisted of 22 cases from 22 patients with a median age of 68 years (55–83) and showed a male predominance (M/F = 2:1). Eight cases were reclassified after centralized review and IHC reassessment: three previously diagnosed as oncocytic renal neoplasms of low malignant potential NOS, five as E-chRCC, and two as LOT. Although oncocytomas are benign and not routinely staged, pT staging was included for comparative purposes. Nineteen cases (86.4%) were confined within the kidney (pT1), three cases (13.6%) were pT3a, and two patients had multiple oncocytic lesions in the same kidney. Macroscopically, oncocytomas also had tan-yellow or brown cut surfaces. Some tumors exhibited central scarring, hemorrhagic areas, or a solid-cystic appearance. There were three main architectural patterns noted: small solid nested (n = 4, 18.2%) or solid/trabecular (n = 3; 13.6%) and tubular/alveolar (n = 15; 68.2%) (Figure 2A–C). All tumors showed granular to densely eosinophilic cytoplasm. Perinuclear halos were noted in 17 cases (77.3%), typically patchy. Thirteen cases (59.1%) had scattered distributed cells with irregular nuclear contours (Figure 2D). CK7 staining was negative in 14/22 cases (63.6%) and focal in the remaining 8 (36.4%) (Figure 2E). CD117 was positive in all tumors, with 15 showing diffuse and 7 showing focal membranous staining (Figure 2F). All cases were negative for GATA 3. Cathepsin K was negative in all but one case, which showed focal staining.

### 3.3. Eosinophilic Variant of Chromophobe Renal Cell Carcinoma Cohort

The E-chRCC cohort consisted of 18 cases from 18 patients with a median age of 63 years (44–85) and showed a slight female predominance (M/F = 1:1.25).

Fifteen cases (83.3%) were confined within the kidney (pT1-2), and three cases (16.7%) were pT3a.

Macroscopically, E-chRCCs exhibited tan-yellow to brown cut surfaces, occasionally with hemorrhagic areas.

**Figure 3:**
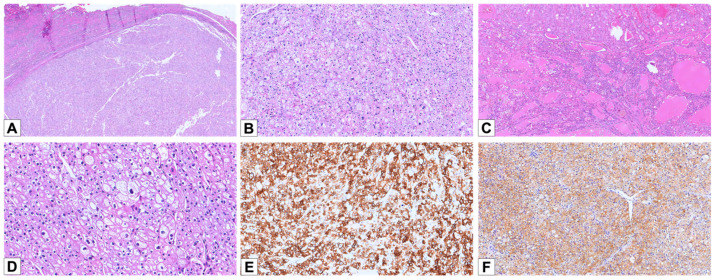
Histopathological and immunohistochemical features of E-chRCC. (**A**) Hematoxylin and eosin staining of a E-chRCC case, demonstrating the characteristic solid/trabecular architecture (HE, 25×). (**B**) The tumor cells exhibit a solid architecture, with slightly eosinophilic cytoplasm and prominent perinuclear halos (HE, 100×). (**C**) A case of E-chRCC showing a prevalent tubular/alveolar architectural pattern (HE, 50×). (**D**) E-chRCC cells typically displaying irregular nuclear contours and prominent perinuclear halos (HE, 200×). (**E**) Diffuse and strong positive staining for CK7 (100×). (**F**) Diffuse cytoplasmic positivity for CD117 (100×).

Only two architectural patterns were observed: solid sheets or broad trabecular arrangements with incomplete vascular septa (annotated as “classic chRCC”) (n = 17; 94.4%) and tubular/alveolar (n = 1; 5.6%) (Figure 3A–C); no cases were noted with the “small nested” pattern. All tumors showed granular to densely eosinophilic cytoplasm with perinuclear halo: in 16 cases, it was diffusely evident, and in 2 cases, it was patchy. All cases showed cells with irregular nuclear contours: in 16 cases, it was widely distributed, and in 2 cases, it was scattered (Figure 3D).

CK7 was positive in all tumors (14 diffuse and 4 focal) (Figure 3E). CD117 was positive in 18/18 cases (100%) (14 cases diffuse and 4 focal) (Figure 3F). All cases were negative for GATA3 and cathepsin K.

### 3.4. Eosinophilic Vacuolated Tumor Cohort

The EVT cohort consisted of four cases from four patients; three cases previously diagnosed as oncocytomas (two cases) and oncocytic renal neoplasm of low malignant potential NOS (one case) were reclassified as EVT following centralized histologic and immunohistochemical reassessment. The median age of patients was 68 years (58–78) at diagnosis, and there was a male predominance (M/F 3:1). All cases were confined within the kidney (pT1).

Grossly, the tumors were well-demarcated and had tan-yellow or brown cut surfaces with hemorrhagic areas; focal cystic changes were seen in one case.

**Figure 4:**
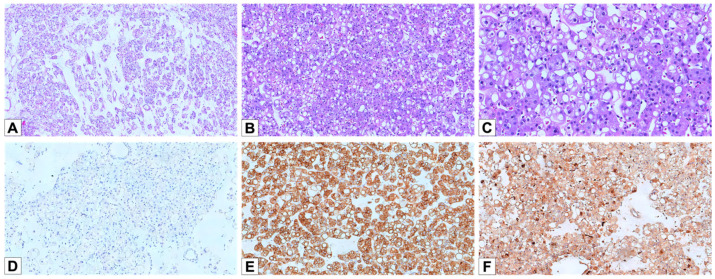
Histopathological and immunohistochemical features of EVT. (**A**) Hematoxylin and eosin staining of an EVT case, demonstrating the characteristic architectural pattern, with tubular/alveolar arrangement and loose edematous stroma (HE, 50×). (**B**) The tumor cells exhibit eosinophilic cytoplasm with prominent intracytoplasmic vacuoles, a hallmark of EVT (HE, 100×). (**C**) The nuclei are often atypical, with irregular contours and distinct, prominent nucleoli (HE, 200×). (**D**) Negative staining for CK7 in EVT cells (100×). (**E**) Strong and diffuse positivity for CD117 (100×). (**F**) Diffuse positivity for Cathepsin K (100×).

Microscopically, three cases (75%) displayed a tubular/alveolar architecture, and one (25%) showed a mixed solid/trabecular type and tubular pattern (Figure 4A,B). Perinuclear halos were observed in 2/4 cases (50%), while all tumors displayed irregular nuclear contours (Figure 4C). One case displayed the typical EVT phenotype, with solid sheets of eosinophilic cells, prominent cytoplasmic vacuolation, and nucleolar prominence. The other three cases presented solid growth with granular and eosinophilic cytoplasm.

CK7 was negative in all cases (Figure 4D). CD117 was diffusely positive in three cases and focal/patchy in one (Figure 4E). Cathepsin K was positive in all tumors (one diffuse and three focal) (Figure 4F). GATA3 was negative in three and focally positive in one case.

### 3.5. L1CAM Expression in All Cohorts

**Figure 5:**
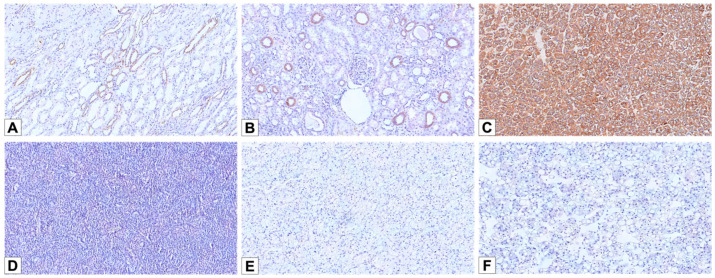
L1 cell adhesion molecule (L1CAM) immunohistochemical stainings. (**A**) L1CAM immunostain labeling the basolateral membrane of the principal cells of medullary collecting ducts (100×). (**B**) A subset of cortical distal tubules showing L1CAM expression (100×). (**C**) Diffuse and strong membranous L1CAM staining in LOT (100×). (**D**–**F**) Negative staining for L1CAM in oncocytoma (**D**), E-chRCC (**E**), and EVT (**F**) cases (100×).

In the normal/background kidney, L1CAM staining was observed predominantly on the basolateral membrane of most medullary collecting duct lining cells (Figure 5A). Among these, scattered cells lacking L1CAM staining were also identified, particularly at the basal aspect, likely representing intercalated cells. In the renal cortex, L1CAM was detected in a subset of distal tubules (Figure 5B).

All 10 LOT cases showed strong membranous L1CAM expression (Figure 5C)—4 patchy and 6 diffuse—including the case with focal CD117 positivity. In contrast, all cases from the other cohorts (oncocytoma, E-chRCC, and EVT) were completely negative for L1CAM (Figure 5D–F). L1CAM showed 100% sensitivity (10/10; 95% CI: 69.2–100%) and 100% specificity (44/44; 95% CI: 92.0–100%) in distinguishing LOT from the other eosinophilic renal neoplasms in this cohort (oncocytoma, E-chRCC, and EVT).

**Figure 6:**
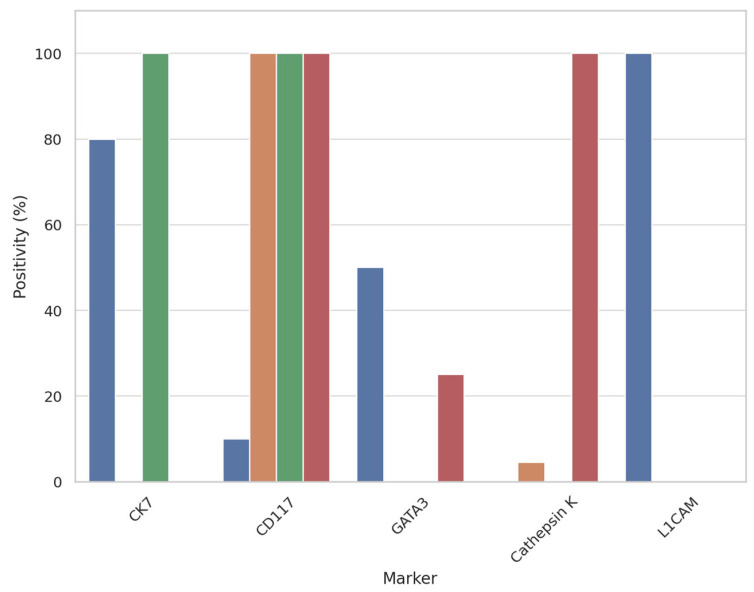
Bar plot showing the immunohistochemical expression rates (%) of CK7, CD117, GATA3, cathepsin K, and L1CAM across four renal tumor types: Low-grade oncocytic tumor (LOT), oncocytoma, eosinophilic chromophobe renal cell carcinoma (E-chRCC), and eosinophilic vacuolated tumor (EVT). L1CAM expression is exclusive to LOT, supporting its diagnostic specificity in this setting.

A graphical summary of immunohistochemical results and marker expression across tumor cohorts is provided in Figure 6.

## 4. Discussion

Accurate differential diagnosis of eosinophilic renal tumors remains challenging due to substantial overlap in morphology and immunophenotype. This difficulty is particularly relevant in small biopsies or when assessed by general pathologists, underlining the need for objective and specific diagnostic markers. The recently identified LOT shares similarities with other eosinophilic renal neoplasms, including E-chRCC, oncocytoma, and EVT. All of these may display eosinophilic cytoplasm, and LOT and E-chRCC, in particular, can have overlapping morphological features, like perinuclear clearing and irregular nuclear contours. While there are subtle differences that could aid in their differentiation, these may not always be easy to identify, particularly in limited biopsy material or when assessed by non-subspecialized pathologists. This complexity underscores the need for additional diagnostic tools, such as immunohistochemical profiling, to support accurate classification. CK7 and CD117 expression patterns vary across LOT, E-chRCC, oncocytoma, and EVT but are not always sufficiently specific to ensure reliable differentiation. Both LOT and E-chRCC often show diffuse CK7 positivity, making it difficult to differentiate them based on this marker alone. Oncocytomas and EVT, on the other hand, tend to be CK7-negative or only show focal expression in a few isolated cells. CD117 is typically negative in LOT but usually positive in E-chRCC, oncocytoma, and EVT, which could help differentiate them. However, due to the variable expression of these markers across different cases and tumor types, they are not definitive for distinguishing these neoplasms [3,9]. Taken together, these immunoprofiles show that traditional markers alone do not offer a reliable way to differentiate LOT from its closest mimickers. For instance, both LOT and E-chRCC are diffusely CK7-positive but differ in CD117 and L1CAM expression, while oncocytoma and EVT may resemble LOT morphologically yet lack both CK7 and L1CAM positivity. This underscores the added value of L1CAM as a diagnostic discriminator.

L1CAM, a marker for the principal cells of collecting ducts [10], has recently emerged as a reliable tool for distinguishing LOT from E-chRCC. L1CAM is consistently positive in LOT, while it is typically negative or weakly expressed in E-chRCC [5]. This marker offers high sensitivity and specificity, making it a valuable asset in differentiating these two morphologically similar tumors. These observations are supported by a recent study by Wang et al. [6], who analyzed a large series of eosinophilic renal tumors, including LOT, oncocytoma, E-chRCC, and oncocytic neoplasms not otherwise specified. Their results showed diffuse membranous L1CAM expression in all LOTs, with negative or focal non-membranous staining in the other tumor types. Notably, their cohort also included Birt–Hogg–Dubé-associated and hybrid oncocytic/chromophobe tumors, expanding the differential context. Compared to their work, our study incorporates eosinophilic vacuolated tumors (EVTs) and applies centralized histopathologic review across multiple centers, strengthening the practical diagnostic value of L1CAM in routine pathology.

Compared to previous studies, our work offers several original contributions. Wang et al. [6] demonstrated diffuse membranous L1CAM expression in LOTs, but their cohort also included hybrid oncocytic/chromophobe tumors and BHD-associated lesions, without addressing EVTs. In contrast, our study incorporates eosinophilic vacuolated tumors (EVT), which have not been previously evaluated for L1CAM expression. Furthermore, unlike earlier reports based on single-institution cohorts, our analysis reflects a centralized histologic and immunohistochemical reassessment across multiple academic centers, increasing diagnostic reproducibility and practical relevance. These aspects strengthen the generalizability and diagnostic applicability of our findings in routine pathology practice.

From a molecular standpoint, the consistent L1CAM expression in LOT may reflect its origin from principal cells of the collecting duct, which are known to express L1CAM physiologically. Recent studies have identified activating mutations in the mTORC1 pathway as a molecular hallmark of LOT, supporting this cell lineage [8,11,12,13]. Interestingly, such mutations are not exclusive to LOT and have also been reported in EVT and other renal tumors with eosinophilic features [14]. Whether L1CAM expression represents a specific transcriptomic signature of principal cells or a broader consequence of mTORC1 activation remains to be clarified. Future integrative studies combining transcriptomic, epigenetic, and immunohistochemical data will be crucial to elucidate this relationship.

Our study focused on assessing the diagnostic performance of L1CAM across a comprehensive cohort of eosinophilic renal tumors, including 10 LOTs, to evaluate its specificity and potential exclusivity in this setting. In addition to evaluating L1CAM expression, we also analyzed and compared the clinical, morphological, and immunohistochemical characteristics of four tumor cohorts from different Italian centers to enhance our understanding of their defining diagnostic features and improve differentiation between them.

Clinically, we found that LOT had a female predominance, in contrast to the male predominance observed in oncocytoma and EVT and no sex predilection in the E-chRCC cohort. The average age was similar across all four groups, with E-chRCC having the lowest (63 years) and LOT the highest (70 years). Tumor sizes were similar across the groups, with LOT having the smallest average size (3.55 cm) and EVT the largest (5 cm). For oncocytoma, LOT, and EVT, pT staging was included for comparative purposes, although its prognostic relevance in benign tumors is limited. pT1 was the most common stage in all groups, with only one case of LOT and one case of E-chRCC at stage pT2 and a few cases of oncocytoma and E-chRCC at stage pT3a. No cases had lymph node or distant metastases.

Morphologically, the LOT group showed a uniform architectural pattern with tightly packed small nests and focal edematous areas in all cases. Only 18.2% of oncocytoma cases had a “small nested” architecture, and neither E-chRCC nor EVT showed this pattern. Oncocytomas primarily exhibited a tubular/alveolar pattern (68.2%), with a smaller proportion showing a solid/trabecular pattern (13.6%). E-chRCC mostly displayed a solid/trabecular pattern (94.4%), while EVT commonly showed a tubular/alveolar pattern (75%). Perinuclear halos were present in all LOT cases (100%) and also in all E-chRCC cases (100%) as well as in many oncocytoma (77.3%) and EVT cases (50%). Irregular nuclear contours were present in all LOT, E-chRCC, and EVT cases and in most oncocytomas (59.1%). These findings suggest that while architectural patterns are helpful, perinuclear halos and irregular nuclear contours are less specific for distinguishing between these entities.

IHC staining revealed limited variation in CK7 and CD117 expression in LOT cases, with only one tumor showing an atypical pattern (CK7 and CD117 diffusely positive). Two LOT cases showed only focal/patchy CK7 staining. However, these cases demonstrated classical LOT morphology, strong and diffuse L1CAM membranous expression, GATA3 positivity, and absence of CD117 and cathepsin K. The diagnosis was therefore supported by the combined morphologic and immunophenotypic profile, consistent with emerging definitions that allow for some variation in CK7 expression among LOTs [11]. Oncocytomas were 100% positive for CD117, though weak and/or focal, while CK7 expression was variable, ranging from negative to weakly positive but never diffuse. In contrast, E-chRCC cases were all diffusely positive for CK7 (100%) and positive for CD117 (100%), though some cases showed weak and/or focal staining. EVT cases were negative for CK7 and positive for CD117, often focal. Regarding other markers, all LOT cases showed nuclear expression of GATA3, but it was consistently focal and of weak to moderate intensity. GATA3 positivity was also observed in one EVT case. Cathepsin K, typically associated with EVT [15,16], was expressed in all EVT cases but also aberrantly in one oncocytoma. These findings underscore the difficulty in differentiating LOT from other eosinophilic renal tumors, even with IHC markers, in addition to morphological criteria.

Although L1CAM showed 100% sensitivity (10/10) and specificity (44/44) in distinguishing LOT from other eosinophilic renal tumors in our series, these results should be interpreted within the context of our cohort size. Confidence intervals calculated using the Clopper–Pearson exact method (sensitivity: 69.2–100%; specificity: 92.0–100%) confirm the excellent performance of this marker while acknowledging the statistical variability inherent to relatively small series. The limited number of EVT cases (n = 4), reflecting the rarity of this entity, is also recognized as a potential limitation. However, the consistency of L1CAM expression across all LOT cases and its complete absence in 44 tumors of different eosinophilic lineages strengthens the diagnostic value of this marker.

L1CAM has proven to be an effective tool in clearly and specifically differentiating LOT from other eosinophilic renal tumors. It not only distinguishes LOT from E-chRCC, as previously shown by Alghamdi et al. [5], but also helps differentiate it from oncocytomas and EVT. L1CAM exhibited distinct staining patterns between the LOT cohort and the other three groups (oncocytoma, E-chRCC, and EVT), making it particularly valuable in challenging cases with unusual histologic or IHC features. In the human kidney, L1CAM is specifically expressed in the ureteric bud, collecting ducts, and distal connecting tubules, with its presence limited to principal cells within the collecting ducts, and not in intercalated cells [17]. Recent single-cell studies have also confirmed L1CAM expression in the principal cell cluster [18,19,20]. Given the strong and diffuse L1CAM staining observed in all LOT tumors but not in E-chRCC, Alghamdi et al. proposed that LOT likely originates from principal cells of the collecting ducts [5]. Our study confirmed this finding, showing that L1CAM was consistently negative in all E-chRCC cases and strongly and diffusely positive in all LOT cases. However, it is worth noting that Alghamdi et al. [5] reported rare cases of E-chRCC and oncocytic neoplasms NOS showing weak or patchy L1CAM immunoreactivity. These cases differed from LOT in both staining pattern and intensity, lacking the strong, diffuse, membranous labeling that characterizes true LOT. Such findings underscore the importance of strict interpretation criteria, focusing on diffuse membranous staining, when using L1CAM as a diagnostic adjunct. Additionally, L1CAM IHC proved useful in distinguishing LOT from renal oncocytoma and EVT. By analyzing L1CAM expression in these other eosinophilic renal tumors, we found that it was completely absent in all cases. Our results confirm, as previously observed by Alghamdi et al. [5], that L1CAM is a specific marker for differentiating LOT from E-chRCC. Furthermore, our study is the first to demonstrate the reliability of L1CAM in distinguishing LOT from other eosinophilic renal tumors, such as oncocytomas and EVT. These findings suggest, consistent with Alghamdi et al.’s observations [5], that LOT may be the only tumor among these to originate from the principal cells of the collecting ducts, while oncocytomas and EVT, as E-chRCC, likely originate from a different cell type, probably intercalated cells. Interestingly, another recently identified indolent renal neoplasm, papillary renal neoplasm of reverse polarity, has been consistently found to exhibit diffuse membranous staining for both L1CAM and GATA3 [21,22,23,24,25]. Whether these similar IHC profiles point to a shared cell of origin between this neoplasm and LOT remains to be further investigated.

However, since L1CAM is upregulated in various cancer types, its role in the broader differential diagnosis of renal cell neoplasms needs further exploration. L1CAM is known to be upregulated in several malignant tumors, where it may contribute to unfavorable outcomes by enhancing tumor cell motility and metastasis [26]. For instance, intense L1CAM expression has been noted in 4% of ccRCC cases, which is associated with shorter survival and downregulation of PAX8 [27]. Moreover, while mTORC1 mutations are a primary driver of LOT [5], they are neither specific nor required for its diagnosis, as they are also found in other renal tumor types, such as EVT, eosinophilic solid and cystic carcinoma (ESC), and renal cell carcinoma with fibromyomatous stroma [28]. Therefore, further investigation into L1CAM expression in other TSC and mTORC1-related tumors is needed. Furthermore, Sangoi et al. recently reported frequent L1CAM expression in succinate dehydrogenase-deficient renal cell carcinomas (SDH-def RCC), with membranous positivity observed in 72% of cases, often with moderate to strong intensity [7]. While these tumors were not included in our cohort, their potential morphologic overlap with LOT, especially in the absence of SDHB immunohistochemistry, raises a relevant diagnostic pitfall. This finding suggests that the specificity of L1CAM may be overestimated when SDH-def RCC is not considered in the differential diagnosis. From a clinical standpoint, accurate classification of these tumors is essential. Misdiagnosing LOT as E-chRCC may lead to unnecessary surveillance or overtreatment, while mistaking an oncocytoma for LOT could underestimate the need for follow-up. The use of L1CAM, particularly in small biopsies or ambiguous cases, may help avoid such pitfalls and improve patient management.

## 5. Conclusions

Our study confirms that LOT is a benign renal neoplasm distinct from other eosinophilic renal tumors, including oncocytoma, eosinophilic chromophobe RCC (E-chRCC), and eosinophilic vacuolated tumor (EVT). Its characteristic morphology—marked by a small nested architecture with perinuclear halos and irregular nuclear contours—can overlap with features seen in other oncocytic tumors, making accurate classification challenging, particularly in small or fragmented biopsies. While LOT typically displays a consistent immunophenotype (diffuse CK7 positivity, CD117 negativity, and focal GATA3 expression), these markers alone may be insufficient to achieve a confident diagnosis in all cases.

In this context, L1CAM emerged in our study as a highly sensitive and relatively specific immunohistochemical marker for LOT. It was diffusely and strongly expressed in all LOT cases and completely absent in E-chRCC, oncocytoma, and EVT, supporting its potential diagnostic value in routine pathology. However, it is important to interpret L1CAM results with caution. Our study, like previous reports [6,29], did not include Birt–Hogg–Dubé-associated tumors, such as hybrid oncocytic/chromophobe neoplasms, which may occasionally express L1CAM. Moreover, recent studies have documented L1CAM positivity in a subset of FLCN-mutated and other eosinophilic renal tumors, suggesting that it is not entirely specific to LOT and may reflect shared differentiation pathways.

The main limitations of our study are the relatively small sample size, particularly for LOT (n = 10) and EVT (n = 4), which reflects the rarity of these entities in clinical practice and the difficulty of collecting larger cohorts even through multicenter collaboration. Nevertheless, the consistent morphologic and immunophenotypic findings observed across institutions reinforce the reproducibility and diagnostic utility of L1CAM in this setting.

Future research should aim to validate these results in larger, independent series and investigate L1CAM expression in other eosinophilic renal neoplasms not addressed in this study, including TSC/mTOR-driven tumors, BHD-associated lesions, and SDH-deficient RCCs. Additionally, integrating L1CAM immunohistochemical profiling with molecular and transcriptomic data may help clarify its lineage specificity, biological role, and potential clinical utility. A better understanding of these aspects could ultimately refine the diagnostic approach to eosinophilic renal tumors and improve clinical decision making, avoiding both over- and undertreatment.

## Figures and Tables

**Table 1:** Criteria used for the immunohistochemical evaluation of the various markers (CK7, CD117, GATA3, cathepsin K, and L1CAM).

IHC Markers	Pattern/Percentage of Cells Stained		
	Positive		Negative
CK7	Diffuse	Focal/Patchy	0−5% cells
CD117	Diffuse	Focal/Patchy	0−5% cells
GATA 3	Diffuse	Focal/Patchy	0−5% cells
Cathepsin K	Diffuse	Focal/Patchy	0−5% cells
L1CAM	Diffuse	Focal/Patchy	0−5% cells

**Table 2:** Summary of clinicopathologic features in LOT, oncocytoma, E-chRCC, and EVT cohorts.

	Cohorts			
	LOT (n = 10)	Oncocytoma (n = 22)	E-chRCC (n = 18)	EVT (n = 4)
***No. of patients***	10	22	18	4
***Age (y*** *)*	70 (58–77)	68 (55–83)	63 (44–85)	68 (58–78)
***Sex***				
Male	1 (10%)	15 (68.2%)	8 (44.4%)	3 (75%)
Female	9 (90%)	7 (31.8%)	10 (55.6%)	1 (25%)
***Tumors size (cm)***	3.55 (1–10)	3.75 (1–8)	4 (1.5–9.5)	5 (2–7)
***Pathologic stage***				
pT1	8 (80%)	19 (86.4%)	14 (77.8%)	4 (100%)
pT2	2 (20%)	/	1 (5.5%)	/
pT3a	/	3 (13.6%)	3 (16.7%)	/
Nx/N0	10 (100%)	22 (100%)	18 (100%)	4 (100%)
M0	10 (100%)	22 (100%)	18 (100%)	4 (100%)
***Predominant pattern***				
Small nested (SN)	10 (100%)	4 (18.2%)	/	/
Solid/trabecular (ST)	/	3 (13.6%)	17 (94.4%)	1 (25%)
Tubular/alveolar (TA)	/	15 (68.2%)	1 (5.6%)	3 (75%)
***Perinuclear halos***				
*Absent*	/	5 (22.7%)	/	2 (50%)
*Present*	10 (100%)	17 (77.3%)	18 (100%)	2 (50%)
***Irregular nuclear contour***				
Absent	/	9 (40.9%)	/	/
Present	10 (100%)	13 (59.1%)	18 (100%)	4 (100%)
***Immunohistochemistry***				
CK7-diffusely positive	8/10 (80%)	0/22 (0%)	18/18 (100%)	0/4 (0%)
CD117-positive	1/10 (10%)	22/22 (100%)	18/18 (100%)	4/4 (100%)
GATA3-diffusely positive	5/10 (50%)	0/22 (0%)	0/18 (0%)	1/4 (25%)
Cathepsin K-positive	0/10 (0%)	1/22 (4.5%)	0/18 (0%)	4/4 (100%)
L1CAM-positive	10/10 (100%)	0/22 (0%)	0/18 (0%)	0/4 (0%)

## Data Availability

The datasets used and/or analyzed during the current study are available from the corresponding author on reasonable request.
